# Pharmacokinetic profiling of rituximab in glomerular diseases: key determinants and implications for personalized therapy

**DOI:** 10.3389/fphar.2026.1778113

**Published:** 2026-04-08

**Authors:** Jingyan Zhang, Qijun Wan, Yuan Cheng

**Affiliations:** Department of Nephrology, Shenzhen Second People’s Hospital, First Affiliated Hospital of Shenzhen University, Shenzhen, China

**Keywords:** glomerulonephritis, pharmacokinetics, rituximab, serum concentration, therapeutic efficacy

## Abstract

Rituximab demonstrates significant efficacy in the treatment of glomerular diseases; however, considerable heterogeneity in clinical responses is observed. This variability is largely attributable to the complex pharmacokinetic profile of rituximab, which is a key determinant of interindividual differences in treatment outcomes. To systematically elucidate the pharmacokinetic characteristics of rituximab across different glomerulopathies and their association with clinical efficacy, this review synthesizes current literature, with a focus on analyzing the impact of key variables, including proteinuria, anti-drug antibodies, and competition for the neonatal Fc receptor, on drug clearance. Furthermore, we compare the dynamic serum concentration profiles and therapeutic outcomes of rituximab in membranous nephropathy, minimal change disease, and lupus nephritis. The findings reveal that the pharmacokinetics of rituximab in patients with glomerular diseases are highly heterogeneous, modulated by both disease-specific factors (for example, damage to the glomerular filtration barrier leads to the urinary loss of proteins) and patient-intrinsic factors (such as polymorphisms in the Neonatal Fc Receptor gene). Available evidence indicates that subtherapeutic drug exposure is closely associated with incomplete B-cell depletion and suboptimal clinical remission. Based on these insights, we identify critical monitoring timepoints for early detection of insufficient exposure (for instance, months 2–3 in membranous nephropathy and month 2 in lupus nephritis). Nevertheless, current data are predominantly derived from retrospective and small-sample studies, and evidence-based target concentration ranges specific to glomerular diseases remain undefined. This review aims to provide an evidence-based rationale and practical recommendations for personalized dosing strategies guided by therapeutic drug monitoring.

## Introduction

Rituximab (RTX), an anti-CD20 monoclonal antibody (Remuzzi et al., 2016), has demonstrated significant therapeutic efficacy in various glomerular diseases through its mechanism of B-cell depletion (Hartinger et al., 2022; Golay et al., 2014; Bondza et al., 2020; Sinha et al., 2021). In membranous nephropathy (MN), RTX has been recommended as a first-line immunosuppressant by KDIGO guidelines (Rovin et al., 2021), and has demonstrated the potential to reduce relapse rates and maintain remission in patients with focal segmental glomerulosclerosis (Lan et al., 2024; Tedesco et al., 2022), minimal change disease (MCD) (Xu et al., 2024; Guan et al., 2023; Fenoglio et al., 2018), and lupus nephritis (LN) (Looney et al., 2004), providing an important therapeutic option for refractory cases.

However, some patients exhibit suboptimal responses to or relapse after RTX treatment. In MN, the clinical/immunological remission rates of RTX are lower in patients with Phospholipase A2 Receptor (PLA2R)-associated MN (Gauckler et al., 2021), with some patients failing to achieve remission even after the first treatment course (Teisseyre et al., 2021a), suggesting that persistent antibody production by long-lived plasma cells may attenuate the therapeutic efficacy of B-cell depletion. Furthermore, epitope spreading may indicate a broader and more established autoimmune response, further limiting treatment efficacy (Seitz-Polski et al., 2018).

Beyond disease-intrinsic factors, drug-related factors are also noteworthy. The development of ADA can neutralize drug activity or reduce serum RTX concentrations (described in detail below), leading to B-cell depletion failure and disease relapse, which is particularly prominent in patients receiving repeated administrations (Ahn et al., 2014) and may be accompanied by severe adverse reactions.

Recent studies have further revealed that low drug exposure is closely associated with suboptimal B-cell depletion and poor clinical remission (Looney et al., 2004; Teisseyre et al., 2021a; Boyer-Suavet et al., 2019). The standard fixed-dose regimen of RTX cannot achieve individualized drug exposure. It fails to address patients’ pharmacokinetic heterogeneity. This variability arises from differences in intrinsic clearance, anti-drug antibody (ADA) formation, and glomerular filtration barrier disruption. Consequently, serum drug levels fluctuate and affect treatment responses. Preliminary evidence links these altered levels primarily to proteinuria, ADAs, and Neonatal Fc Receptor (FcRn)-related competition. However, these insights lack a systematic horizontal integration of pharmacokinetic profiles across different glomerular disease contexts and have not yet evolved into a clear, treatment-drug-monitoring-based framework for guiding clinical decision-making.

Therefore, this review synthesizes evidence regarding the factors influencing the pharmacokinetics of RTX, its pharmacokinetic characteristics and efficacy in patients with different glomerular diseases, and identifies optimal time points for therapeutic drug monitoring. This analysis aims to provide a theoretical foundation and practical guidance for optimizing the clinical application of RTX.

## Methods

### Literature search strategy

A systematic literature search was conducted on the PubMed database, covering its inception up to December 2025. The main search terms and their combinations included: “Rituximab” AND (“glomerular disease” OR “nephropathy” OR “membranous nephropathy” OR “minimal change disease” OR “lupus nephritis”) AND (“pharmacokinetics” OR “clearance” OR “drug exposure” OR “half-life”). The search was restricted to English-language publications.

### Inclusion and exclusion criteria

This review included original research (comprising clinical trials, cohort studies, retrospective studies, and case series), review articles, and expert consensus that discussed rituximab pharmacokinetic properties, influencing factors, or pharmacodynamic correlations in patients with glomerular diseases. Exclusion criteria were studies irrelevant to the topic, incomplete data, non-human studies, and non-English publications.

### Study selection process

Search results were initially screened by title and abstract to exclude clearly irrelevant articles. Full-text articles of the remaining literature were retrieved and independently assessed by two researchers (Jingyan Zhang and Yuan Cheng) to determine their eligibility against the inclusion criteria. Any discrepancies were resolved through discussion. Finally, the included literature was used for data extraction and synthesis. This review intends to provide an overview of the existing literature rather than a systematic meta-analysis.

### Data extraction and synthesis

From each included study, key information was extracted, including study design, sample size, patient characteristics, rituximab dosage and administration regimens, pharmacokinetic parameters (e.g.,.half-life, clearance), pharmacodynamic outcomes (e.g., B-cell depletion, antibody levels), and factors influencing rituximab clearance (e.g., ADA), proteinuria, FcRn, concomitant medications). The extracted data were synthesized through qualitative description and thematic analysis to identify key findings, trends, and unresolved questions. During the summarization process, the quality and potential limitations of the cited studies were also considered.

## Key determinants of RTX blood concentration

After intravenous administration, RTX rapidly distributes into plasma and extracellular fluid, with a steady-state volume of distribution of 3.0–9.6 L (Hartinger et al., 2022; Golay et al., 2014). Its clearance is non-linear, primarily occurring through binding to CD20 on B-cell surfaces, mediating complement-dependent cytotoxicity and antibody-dependent cellular phagocytosis, followed by internalization and lysosomal degradation (Hartinger et al., 2022; Golay et al., 2014; Sinha et al., 2021). During this process, the FcRn-mediated recycling mechanism significantly prolongs its half-life, while unprotected antibodies are rapidly cleared via the lysosomal pathway (Golay et al., 2014).

Based on the aforementioned mechanisms, variations in RTX blood concentration are primarily determined by its clearance rate. Therefore, identifying and controlling factors that influence its clearance is crucial for maintaining effective drug concentrations. Key determinants of clearance include target-mediated drug disposition, FcRn-mediated protection, and non-specific clearance.

### FcRn and IgG

FcRn is a critical regulator of Immunoglobulin G (IgG) antibody persistence *in vivo*. It significantly prolongs the half-life of IgG and reduces its lysosomal degradation by mediating antibody recycling in an acidic environment, thereby maintaining stable therapeutic antibody concentrations (Hartinger et al., 2022; Golay et al., 2014; Pyzik et al., 2023). In FcRn-deficient animal models, the clearance of monoclonal IgG antibodies increases by 10–15-fold (Garg and Balthasar, 2007). Notably, FcRn expression exhibits significant interindividual variability and genetic polymorphism (Garg and Balthasar, 2007; Fogueri et al., 2018; Israel et al., 1996). Studies have shown that in some patients, residual drug can still be detected 6–9 months after the last RTX infusion, suggesting that elevated FcRn expression levels or binding affinity may lead to delayed RTX clearance and increased drug exposure (Boye et al., 2003). Such patients might derive limited benefit from additional RTX doses if they show a poor response to initial treatment.

Simultaneously, endogenous IgG levels constitute a significant competitive factor influencing RTX pharmacokinetics. Studies in patients with rheumatoid arthritis have confirmed a strong positive correlation (P = 7.4 × 10^−8^) between serum IgG concentration and the RTX clearance rate constant (k_10_) (Couderc et al., 2013). This may be attributed to the fact that excessive circulating endogenous IgG competitively occupies the limited FcRn binding sites, thereby attenuating FcRn-mediated protection of RTX, which leads to accelerated RTX clearance, shortened half-life, and reduced serum drug concentrations (Hartinger et al., 2022). The precise quantitative model of FcRn-mediated antibody recycling kinetics, the competition from endogenous IgG, and its value for efficacy prediction remain to be further elucidated. It is recommended to routinely measure patients’ serum IgG levels before initiating RTX therapy. If a significant elevation is detected, active investigation and management of potential underlying causes (such as infection or active autoimmune disease) should be pursued, as this may help optimize RTX pharmacokinetics and thereby improve treatment outcomes.

### ADAs

ADAs are generated as an immune response to exogenous monoclonal antibody therapies (Hartinger et al., 2022; Chirmule et al., 2012; Mok, 2013). The incidence of ADA varies markedly across different glomerular diseases (0%–66%) (Fogueri et al., 2018; Iijima et al., 2014; Vaisman-Mentesh et al., 2020). This variation, aside from methodological differences in detection (Hartinger et al., 2022), suggests that distinct immunological states may influence ADA development. ADA compromises efficacy by accelerating RTX clearance and reducing serum drug concentrations, and may even enhance drug elimination—without interfering with Cluster of Differentiation 20(CD20) binding—through mechanisms such as immune complex formation (Hartinger et al., 2024; Boyer-Suavet et al., 2020). I. Oomen et al. (Oomen et al., 2022) reported that in ADA-positive pediatric patients, RTX concentrations fell below the detection limit within 105 days after administration. Another study in systemic lupus erythematosus (Looney et al., 2004) found that higher ADA titers were associated with significantly reduced RTX levels. This indicates that across various autoimmune diseases, ADAs consistently impair therapeutic efficacy by shortening the *in vivo* persistence of RTX. Additionally, the surface charge characteristics of the antibody molecule may influence its clearance rate, with an increase in positive charge correlating with enhanced clearance (Kamath, 2016).

When ADA leads to B-cell depletion failure (Ahn et al., 2014; Anolik et al., 2004; Md Yusof et al., 2017), besides supplementary dosing to overcome resistance in patients remaining antibody-positive 6 months after initial therapy (Dahan et al., 2019), switching to novel Type II humanized anti-CD20 monoclonal antibodies with distinct spatial structures and glycosylation (e.g., obinutuzumab) is also a viable clinical option (Gagez and Cartron, 2014; Crickx et al., 2020; Furie et al., 2022).

Compared to RTX, obinutuzumab’s humanized design helps minimize the risk of cross-reactivity. Additionally, the afucosylation of its Fc region (Hartinger et al., 2022) amplifies its antibody-dependent cellular cytotoxicity by 10- to 100-fold (Golay et al., 2014). Clinical evidence demonstrates that these enhanced pharmacodynamic properties translate into tangible clinical benefits for refractory patients.

In a comparative study of refractory MN, obinutuzumab achieved a 90.0% overall clinical remission rate (vs. 38.7% with RTX), alongside a significantly higher 6-month immunological remission rate (87.5% vs. 21.4%) (Xu et al., 2025). Notably, in subgroup analyses of patients with confirmed RTX resistance, switching to obinutuzumab yielded clinical remission rates ranging from 80% (8/10 cases (Xu et al., 2025) and 16/20 cases (Su et al., 2025)) to a striking 100% (12/12 cases) (Lin et al., 2024) across distinct cohorts. Furthermore, in pediatric frequently relapsing or steroid-dependent nephrotic syndrome, a single low-dose obinutuzumab infusion (300 mg/1.73 m^2^) effectively depletes peripheral B cells, yielding a 12-month sustained relapse-free remission rate of 92% (Dossier CB et al., 2023). By achieving profound target cell depletion, obinutuzumab serves as an efficacious alternative for improving clinical outcomes in patients with ADA positivity and multidrug resistance.

### Glomerular filtration barrier disruption and urinary drug loss

Beyond immune-mediated targeted clearance mechanisms, local renal pathophysiological alterations act as key factors affecting the pharmacokinetics of RTX. Under normal physiological conditions, the kidney is not the primary route of elimination for large monoclonal antibodies. However, in glomerular diseases, structural disruption of the glomerular filtration barrier leads to a pathologically increased permeability to macromolecules like RTX (Teisseyre et al., 2021b). Severe non-selective proteinuria serves as a direct clinical manifestation of the breakdown of this size-selective barrier (Teisseyre et al., 2021b). This accounts for the high concentrations of RTX detected in the urine of patients with nephrotic syndrome (Jacobs et al., 2017; Stahl et al., 2017). Furthermore, hypoalbuminemia concomitant with barrier disruption alters the drug’s volume of distribution and protein binding. These changes indirectly accelerate RTX clearance, ultimately resulting in suboptimal systemic drug exposure (Golay et al., 2014). A small study on MN demonstrated that the urinary protein excretion rate, an indicator of barrier disruption severity, was significantly and negatively correlated with serum RTX levels (y = −0.0039x + 76.0, *r* ^2^ = 0.1989, p = 0.0488) (Fogueri et al., 2018). Building upon this, Teisseyre M et al. further expanded the sample size to 68 MN patients and observed that a proteinuria level exceeding 5.5 g/day at month 3 was associated with RTX concentrations below 2 μg/mL at that time (Teisseyre et al., 2021b). These findings demonstrate that the urinary leakage of large monoclonal antibodies, driven by a compromised filtration barrier, is a key factor attenuating RTX efficacy.

To mitigate hyperpermeability-induced RTX loss, in addition to considering the concomitant use of angiotensin-converting enzyme inhibitors or angiotensin receptor blockers to improve intra-glomerular hemodynamics, some studies have proposed adding calcineurin inhibitors to RTX therapy. These agents may reduce the urinary loss of RTX by constricting both the afferent and efferent glomerular arterioles (Waldman et al., 2016). However, this approach may increase the risk of infection due to excessive immunosuppression, and its safety profile still requires validation through well-designed multicenter prospective cohort studies.

### Plasmapheresis and dialysis

Plasmapheresis and dialysis represent two special extracorporeal pathways for RTX clearance, both of which significantly reduce its serum concentration (Hartinger et al., 2022; Golay et al., 2014). Plasmapheresis directly removes RTX from the plasma, leading to a sharp decline in drug concentration due to the similar physicochemical properties of RTX and natural antibodies. Several case reports have observed that performing plasmapheresis within 24–36 h after RTX infusion may compromise drug exposure (McDonald et al., 2010; Darabi and Berg, 2006). Puisset et al., through multiple observational and modeling analyses, demonstrated that plasmapheresis conducted 24–72 h after RTX administration could remove 47%–59% of the initial dose in the first session, with clearance negatively correlated to the infusion–exchange interval (r = 0.96). If three consecutive exchanges are performed post-infusion (at 24, 48, and 72 h), the overall area under the concentration–time curve is reduced by approximately 36% (Puisset et al., 2013).

Therefore, it is not recommended to initiate plasmapheresis too shortly after RTX infusion. When unavoidable, adding a third RTX injection following the first plasmapheresis session has been proposed, which could increase overall drug exposure by approximately 13% (Puisset et al., 2013).

Peritoneal dialysis may also increase RTX clearance, particularly in patients with high peritoneal transport or inflammatory states, where RTX can “leak” into the dialysate along with protein (Hartinger et al., 2022). A case report indicated that RTX concentration in peritoneal dialysate could reach 3.5 μg/mL (Stahl et al., 2017). For such patients, vigilance against subtherapeutic drug exposure is warranted. Therapeutic drug monitoring (e.g., trough RTX concentration measurement) is recommended to guide clinical decision-making, with consideration for increased dosing frequency or dose adjustments as needed to maintain effective serum drug levels. In the future, exploring more stable administration routes, such as subcutaneous delivery, in this patient population merits further investigation.

Furthermore, RTX blood concentrations are also influenced by various other factors. Studies have demonstrated that RTX serum concentrations are inversely correlated with target antigen load (Hartinger et al., 2022; Golay et al., 2014) and tumor cell count (Harjunpää et al., 2003; Cartron et al., 2007; Tout et al., 2017), but positively correlated with body surface area (Hartinger et al., 2024). Systemic inflammation (Hartinger et al., 2022) can enhance the nonspecific clearance of RTX, while premenopausal female patients exhibit lower RTX clearance compared to males (Golay et al., 2014; Cornec et al., 2018). These factors should be considered prior to RTX administration.

**FIGURE 1:**
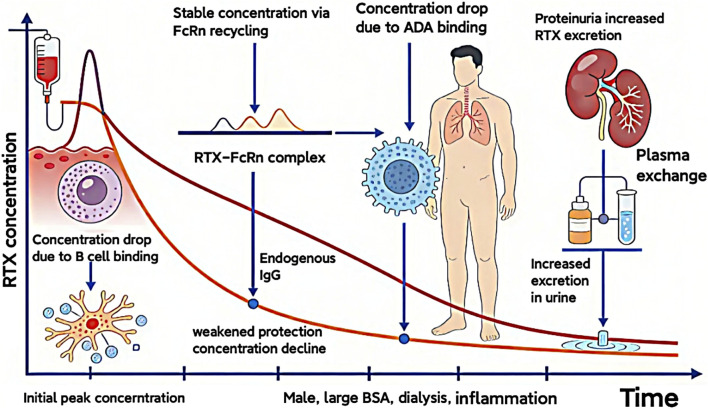
Factors influencing the blood concentration of RTX.

RTX is governed not by an isolated determinant, but by an interconnected pharmacokinetic network involving disease-specific immunological activity (which modulates target antigen burden and the risk of ADA development), direct renal sequelae (notably proteinuria), and patient-specific factors (including polymorphisms in the FcRn) (Figure 1). In clinical practice, concomitant high-grade proteinuria and elevated ADA titers are frequently observed, potentially exerting a synergistic effect that accelerates drug elimination. This combined clearance mechanism may constitute a key pharmacokinetic foundation underlying treatment refractoriness in a subset of patients.

## Serum drug concentration profiles and clinical outcomes in glomerular diseases

The pharmacokinetic properties and therapeutic efficacy of RTX exhibit both commonalities and distinct variations across different glomerular diseases. The subsequent sections will comprehensively discuss the plasma concentration profiles of RTX and their corresponding clinical outcomes in various glomerulopathies, providing valuable clinical insights for treatment optimization.

### MN

The pharmacokinetics of RTX in MN patients are complex and variable, with blood concentrations exhibiting unique dynamic changes over time under different dosing regimens (Table 1). Early studies on the “1g RTX infusion every 2 weeks × 2″regimen (Fervenza et al., 2010) preliminarily revealed that RTX blood concentrations in MN patients significantly decreased by day 15 and were generally lower than in non-proteinuric patients. However, that study included only 15 patients, a small sample size that introduces potential randomness and bias, and it failed to further track concentration changes after the second administration. Under the same 1g × 2 regimen, Seitz-Polski et al. (2019) detected an RTX concentration of only 3.3 μg/mL 3 months post-administration; whereas Boyer-Suavet et al. (2019), through more frequent measurements, found that the blood concentration could reach 47 μg/mL in the first month post-administration but dropped below the limit of detection by the sixth month. This not only highlights the detection discrepancies among different studies but also indicates the rapid decay characteristics of RTX blood concentration under this regimen.

**TABLE 1:** The dynamic changes of RTX blood concentration in MN and its prognosis.

Author	Sample size	RTX regimen	Study design	Test time	Result	Prognosis
FC Fervenza et al. (2008)	15	1g × 2 doses, at d1 and d15	Prospective	1 day	218.6 ± 67 μg/mL	relief rate: 57% (12 months)
				15 days before injection	17 ± 11 μg/mL	
				15 days after injection	205.2 ± 111 μg/mL	
Sonia Boyer-Suavet et al. (2019)	43	1g × 2 doses, at d1 and d15	Prospective	1month	47 μg/mL	relief rate: 57% (39 months)
				3 months	12.7 μg/mL	
				6 months	<2 μg/mL	
Barbara Seitz-Polski et al. (2019)	28	1g × 2 doses, at d1 and d15	Retrospective	3 months	3.3 μg/mL	relief rate: 57% (6 months)
Barbara Seitz-Polski et al. (2019)	27	375 mg/m^2^/week×2 doses	Retrospective	3 months	0 μg/mL	relief rate: 30% (6 months)
Uma Fogueri et al. (2018)	20	375 mg/m^2^/week × 4 doses.; Repeat at the sixth month	Retrospective	0 days	120 μg/mL	—
				1 month	160 μg/mL	
				2 months	70 μg/mL	
				3 months	20 μg/mL	
				4 months	0 μg/mL	
				6 months	130 μg/mL	
				200days	180 μg/mL	
				250days	60 μg/mL	
				1 year	0 μg/mL	
FC Fervenza et al. (2010)	20	375 mg/m^2^/week × 4 doses; Repeat at the sixth month	Prospective	1 day	200 ± 34 μg/mL	relief rate: 88% (24 months)
				8 days before injection	54.7 ± 23.6 μg/mL	
				8 days after injection	229 ± 49.9 μg/mL	
				15 days before injection	88.5 ± 35.4 μg/mL	
				15 days after injection	247 ± 62.7 μg/mL	
				22 days before injection	101 ± 46.2 μg/mL	
				22 days after injection	257 ± 71.5 μg/mL	

Exploration of various dosing regimens has revealed that the “375 mg/m^2^ × 4”regimen demonstrated elevated plasma drug concentrations within the initial 2 months. The pharmacodynamic exposure window for this regimen extended for approximately 6 months. Notably, upon repeat treatment at 6 months, plasma drug concentrations during the first month of the repeated course were significantly higher than those observed during the corresponding period of the initial course (Fogueri et al., 2018), suggesting potential cumulative effects or alterations in B-cell burden. In contrast, with the “infusion of 375 mg/m^2^ × 2” regimen (Seitz-Polski et al., 2019), plasma drug concentrations in patients were generally undetectable by the third month post-administration, indicating a potentially shorter window for maintaining effective exposure. Consequently, earlier and more intensive pharmacokinetic monitoring is warranted for this regimen (e.g., during the first week, second week, and first month of treatment).

However, most of the aforementioned studies involved small sample cohorts, which severely limits the generalizability and extrapolability of their findings. Furthermore, significant differences in confounding factors such as baseline B-cell burden and immune status among patients across different studies make direct comparison of RTX concentration data to infer optimal detection timing challenging. Even though all studies utilized Enzyme-linked Immunosorbent Assay (ELISA) for RTX concentration measurement, variations in the specificity, sensitivity, and calibration of different assay kits undoubtedly influence the absolute values of drug concentrations.

The accurate measurement of serum RTX concentrations is fundamental to pharmacokinetic and exposure-response relationship studies; however, the primary source of data heterogeneity is the intrinsic difference in the principles of various detection methods. This discrepancy arises mainly because each method quantifies different molecular forms of RTX. A monoclonal anti-idiotype ELISA has a lower limit of quantification as low as 0.3 μg/mL (Cartron et al., 2007); a novel peptide-based ELISA using a synthetic cyclic CD20 mimetic peptide has an lower limit of quantification of 0.2 μg/mL (Blas et al., 2007); and the lower limit of quantification for flow cytometry ranges from 0.1 to 1 μg/mL (Cartron et al., 2007). In contrast, a fluoroimmunoassay significantly reduces interference from the complex serum matrix, achieving an lower limit of quantification of 0.09 μg/mL (Liu et al., 2012) (Table 2). All of the above methods can only measure free RTX, because the binding of RTX to its target antigen masks the idiotype binding sites.

**TABLE 2:** Different RTX detection methods and sensitivity.

Method	Detect the type of antibody	Sensibility
Anti-idiotype Sandwich ELISA (Cartron et al., 2007)	Free RTX	0.3 μg/mL
Gyrolab™ Automated Microfluidic Fluorescence Immunoassay (Liu et al., 2012)	Free RTX	0.09 μg/mL
Peptide-based ELISA (Blas et al., 2007)	Free RTX	0.2 μg/mL
The novel anti-RTX unique type antibody 1–11A of mice (using flow cytometry) (Cragg et al., 2004)	Free RTX and RTX that has been attached to the surface of the cells	—
Complement-dependent Cytotoxicity Assay (Beum et al., 2004)	The RTX that has been attached to the cell surface	1 μg/mL

In contrast, the complement-dependent cytotoxicity assay is a functional test that evaluates the biological activity of RTX to induce complement-mediated killing, rather than its mere physical concentration (Beum et al., 2004). To further complicate matters, Cragg et al. (2004) demonstrated that certain specific anti-idiotype antibodies can simultaneously recognize both free and partially bound RTX (Table 2).

This methodological stratification and inconsistency provide a critical analytical perspective for interpreting clinical data. For instance, the significant difference in serum drug concentrations at month 3 observed between the studies by Boyer-Suavet et al. (2019), Seitz-Polski et al. (2018) (12.7 μg/mL vs. 3.3 μg/mL, respectively) is highly likely not due to pharmacokinetic differences, but is instead a measurement artifact. The focus of future research should shift towards establishing a standardized quantification guideline that stipulates all reports must specify the molecular form of RTX being measured (e.g., free or functionally active), thereby providing a uniform benchmark for cross-study data comparison.

Despite these disparities, these studies collectively indicate a general substantial decline in RTX plasma concentrations after the third month of treatment. Given that an *in vitro* study suggested a minimum RTX plasma concentration of approximately 10 μg/mL required for complement-dependent cytotoxicity (Fenoglio et al., 2018; McDonald et al., 2010), this underscores the clinical significance of monitoring plasma drug concentrations during the second to third month of treatment. This measure is crucial for identifying insufficient drug exposure, predicting treatment efficacy, and guiding timely interventions.

Comparative data from different dosing regimens reveal a complex association, rather than a simple linear relationship, between the pharmacokinetic characteristics of RTX (duration of plasma concentration maintenance) and clinical remission rates (Table 1). One study (Seitz-Polski et al., 2018) demonstrated that a high-dose RTX regimen (two 1 g doses, 2 weeks apart) achieved higher circulating RTX levels at 3 months post-treatment (median 3.3 mg/L [0.0–10.8]) and significantly outperformed a low-dose regimen (two 375 mg/m^2^ doses, 1 week apart) in inducing more profound B-cell depletion (median CD19 count 0.0 [0.0–2.0] vs. 16.5 [2.5–31.0]) and a more significant reduction in anti-PLA2R1 antibody levels (median 0.0 [0.0–8.0] vs. 8.3 [0.0–73.5]). These findings strongly suggest that adequate drug exposure more effectively clears pathogenic autoantibodies. Furthermore, higher RTX plasma concentrations at 3 months of treatment (median 2.2 mg/mL [0.0–10.9]) were significantly positively correlated with clinical remission at 6 months (P < 0.001), further quantifying the strong link between drug exposure and clinical outcomes.

Although exposure is positively correlated with efficacy, study results do not always simply point to “the higher the starting dose, the better.” Fernando C. Fervenza et al. compared the “two 1 g doses, 2 weeks apart” regimen (Fervenza et al., 2008) with the “375 mg/m^2^ × 4, 1 week apart” regimen (Fervenza et al., 2010) and found that, despite the latter having a relatively lower initial concentration, it induced more significant B-cell depletion. This suggests that the key to RTX efficacy might lie in its ‘sustained exposure’ within the body. More frequent but lower-dose maintenance therapy may achieve more prolonged and thorough B-cell depletion by preventing RTX levels from falling below a critical threshold during dosing intervals (Hartinger et al., 2022).

Studies have found that RTX treatment can lead to adverse reactions such as hypogammaglobulinemia and neurological disorders (Kaegi et al., 2019; Beum et al., 2011). If drug concentrations acutely increase within a short period, it may lead to rapid and excessive binding of CD20 antigens on the B-cell surface (Golay et al., 2014), thereby accelerating the Fc receptor-positive effector cell-mediated Trogocytosis process (Beum et al., 2006). This phenomenon of “antigen stripping” involves intercellular transfer of membrane material, where effector cells recognize and bind to the RTX-CD20 immune complexes, “nibble” or “capture” their membrane fragments, and internalize them (Liu et al., 2012). This process leads to a rapid decline in CD20 expression on the B-cell surface within a short time, potentially resulting in a “target deficit” situation even before effective B-cell clearance. Consequently, this can reduce overall therapeutic efficacy and even promote the development of functional resistance (Beum et al., 2006). Therefore, the value of therapeutic drug monitoring lies not only in ensuring minimal effective exposure to prevent treatment failure but also in avoiding unnecessary toxicity or potential resistance risks caused by excessive exposure, thereby achieving precise management of the therapeutic window.

### MCD

In the treatment of MCD, although international guidelines have not yet routinely recommended RTX, it has demonstrated clinical potential in refractory patients. Its mechanisms include depleting B cells that secrete harmful cytokines (e.g., B-cell Activating Factor, A Proliferation-Inducing Ligand), inhibiting B-cell mediated T-cell activation to modulate immune balance, and potentially directly stabilizing podocyte structure and function, thereby alleviating podocyte damage and improving the renal immune microenvironment (Zhong et al., 2025; Hansrivijit et al., 2020).

High plasma drug concentrations can achieve sustained clearance of circulating B cells (Berinstein et al., 1998). From a pharmacokinetic perspective, following a single dose (375 mg/m^2^), plasma concentrations rapidly peak (approximately 220 μg/mL) and then exhibit a biphasic decline, with an elimination half-life of about 14.6 days and an average area under the curve of 83.2 mg·h/mL (Kamei et al., 2009). Notably, multi-dose regimens (once weekly × 4 weeks) significantly increase plasma concentrations during treatment (reaching 156 μg/mL before the 4th infusion) and prolong the half-life through cumulative effects. This primarily appears to manifest as enhanced sustained effective exposure, rather than merely a substantial exceedance of peak concentrations. Pharmacodynamic studies further confirm a strong correlation between RTX plasma concentrations and the degree of B-cell depletion; peripheral blood B cells are rapidly depleted to CD19^+^ <5 cells/μL after administration, and this depleted state typically lasts for 119–148 days (Iijima et al., 2014) (Table 3).

**TABLE 3:** Dynamic serum concentration changes, and clinical prognosis of RTX in patients with MCD.

Author	Sample size	RTX regimen	Study design	Test time	Result	Prognosis
Koichi Kamei et al. (2009)	12	375 mg/m^2^/week × 1 dose	Prospective	1 day	166.2 ± 56.2 μg/mL	The complete depletion rate of B cells: 88%; high recurrence rate
				1 week	92.5 ± 29.8 μg/mL	
				1 month	27.4 ± 18.4 μg/mL	
				2 months	18.5 ± 29.8 μg/mL	
				3 months	3.0 ± 4.4 μg/mL	
				5 months	<0.5 μg/mL	
Kazumoto Iijima et al. (2014)	52	75 mg/m^2^/week × 4 doses (up to 500 mg)	Randomized Controlled Trial	22 days	156 μg/mL	Treatment failure-free rate 97.4% at 337 days 84.6% at 505 days
				85 days	28.8 μg/mL	
				169 days	2.32 μg/mL	
				365 days	0 μg/mL	

The duration of pharmacodynamic effect is positively correlated with the area under the curve and the duration of B-cell depletion. Multi-dose regimens (Iijima et al., 2014), by providing higher and more sustained plasma concentrations, extend the B-cell depletion period, thereby significantly prolonging patients’ relapse-free survival (median 267 days vs. 129 days for single-dose). This collectively suggests a strong correlation between relapse and B-cell repopulation. When B cells repopulate, it may lead to the re-initiation of pathogenic immune responses, a significant decline in regulatory T cells, and altered Interleukin-2 expression (Sinha et al., 2021; Zhong et al., 2025). Although these are plausible pathophysiological hypotheses, their precise causal relationships and specific molecular mechanisms still require in-depth elucidation through prospective cohort studies, combined with detailed immune cell subpopulation analysis and cytokine profile monitoring.

The aforementioned studies were small-sample investigations focusing on children from the Japanese region, with limitations such as short follow-up periods and a lack of subgroup discussions. However, they provide valuable insights for the application of RTX in MCD. Based on these studies, we recommend monitoring RTX concentrations at months 2-3 for patients receiving single-dose regimens, and enhancing monitoring at months 4-6 for those on multi-dose regimens. Concurrently, the safe drug concentration has not yet been clearly defined. In rare instances, severe thrombocytopenia (low platelet count) and coagulopathy (impaired clotting function) (Zhong et al., 2025) have been reported. Future research should integrate pharmacokinetic/pharmacodynamic models with clinical outcomes to precisely determine the optimal therapeutic window and individualized concentration thresholds for MCD patients, thereby improving the generalizability and accuracy of these thresholds.

Compared to MN, the half-life of RTX in MCD appears to be slightly longer. This is primarily attributed to their completely distinct pathophysiological mechanisms. In MN, characteristic subepithelial immune complex deposits alter glomerular filtration barrier permeability, accelerating the heavy urinary loss of large proteins like RTX (Dantas et al., 2023). Additionally, the increased susceptibility of MN patients to ADA formation post-RTX treatment (Hartinger et al., 2022; Golay et al., 2014; Teisseyre et al., 2021a) further expedites *in vivo* drug clearance.

In contrast, the pathogenic and recovery mechanisms of MCD present a starkly different paradigm. Emerging evidence reveals that autoantibodies specifically bind to Nephrin, a core protein of the podocyte slit diaphragm, triggering its aberrant phosphorylation and subsequent cytoskeletal rearrangement. These biochemical alterations provoke profound and rapid endocytosis of Nephrin, directly compromising the slit diaphragm. Concomitant with extensive foot process effacement, the physical filtration barrier rapidly collapses, precipitating acute and massive proteinuria (Zhong et al., 2025). In this pathological context, RTX precisely targets and depletes the B cells responsible for producing anti-Nephrin autoantibodies. Following the substantial decline in circulating pathogenic antibodies, slit diaphragm function is swiftly restored, leading to the rapid resolution of proteinuria. Crucially, this early functional recovery of the filtration barrier dramatically minimizes the secondary urinary loss of RTX, thereby effectively prolonging its half-life in patients with MCD.

Currently, there is a lack of direct comparative studies on the pharmacokinetic profiles of RTX in MCD versus MN patients. To clarify the specific mechanisms underlying the differences in RTX pharmacokinetics between these two kidney disease types, it is imperative to conduct multicenter, large-sample comparative studies that also thoroughly evaluate ADA generation and its specific contribution to RTX clearance mechanisms in different nephropathies.

### LN

LN is a complex autoimmune disease. Multiple studies have demonstrated the favorable efficacy of RTX in the treatment of LN (Yuan et al., 2020; Parikh et al., 2020; Cervera et al., 2019). The mechanism of action of RTX lies in its ability to continuously inhibit B cells (Zisa et al., 2024), thereby reducing the production of pathogenic autoantibodies (e.g., Anti-double-stranded DNA) and blocking antigen-presenting cell function. This, in turn, suppresses T-cell activation and lowers the levels of proinflammatory cytokines and chemokines (Yuan et al., 2020). These synergistic effects effectively mitigate the deposition of immune complexes in the kidneys and reduce inflammatory responses, thereby comprehensively suppressing the autoimmune process in LN.

Compared to MN and MCD, LN patients exhibit more rapid RTX clearance. A Phase I/II dose-escalation study, which included only 17 Systemic Lupus Erythematosus patients (7 of whom had Lupus Nephritis), found that the average RTX plasma concentrations had dropped to extremely low levels by 2 months post-administration, regardless of a single dose of 100 mg/m^2^ or 375 mg/m^2^ (Looney et al., 2004). Even with a “4 times weekly 375 mg/m^2^″ regimen, the concurrent average concentration could be maintained at 9.4 μg/mL, which was still below 10 μg/mL. The high B-cell burden present in LN patients, where a large number of CD20-expressing B cells extensively bind and consume circulating RTX via an “antigen sink effect,” leads to rapid drug clearance and a swift decline in plasma concentrations (Hartinger et al., 2022; Golay et al., 2014). Furthermore, this study identified that 6 patients developed ADA, which bind to RTX to form immune complexes, further accelerating its elimination from the body and thereby affecting drug concentration and therapeutic efficacy (Hartinger et al., 2024). It is also noteworthy that some patients concurrently received other immunosuppressants (e.g., corticosteroids, methotrexate, mycophenolate mofetil, and prednisone), and drug interactions between these concomitant medications and RTX cannot be excluded, potentially further influencing RTX pharmacokinetics (Table 4).

**TABLE 4:** Dynamic serum concentration changes, and clinical prognosis of RTX in patients with LN.

Author	Sample Size	RTX Regimen	Study design	Test time	Result	Prognosis
R. John Looney et al. (2004)	18	100 mg/m^2^/week × 1 dose 375 mg/m^2^/week × 1 dose 375 mg/m^2^/week × 4 doses	Retrospective	2 months	0.1 μg/mL 0.32 μg/mL 9.4 μg/mL	Deep B-cell depletion (CD19^+^ <5/μL): 65%

Therefore, maintaining adequate RTX plasma concentrations may be crucial for achieving disease control in refractory autoimmune diseases. Based on the aforementioned research, monitoring RTX plasma concentrations before the second month post-administration is a critical point for evaluating whether treatment intensity is sufficient and for determining the necessity of supplementary dosing.

Unfortunately, this study did not perform subgroup analysis for Systemic Lupus Erythematosus and LN, thus failing to distinguish which factor—intrinsic disease inflammation, ADA generation, or proteinuria associated with renal injury—is the dominant contributor to reduced RTX plasma concentrations, nor did it elucidate their potential interactions. Furthermore, due to the small sample size and inconsistent monitoring time points in this study, future prospective, large-sample pharmacokinetic/pharmacodynamic studies specifically targeting LN are required. These studies should also conduct subgroup analyses for potential influencing factors such as proteinuria and ADA, and simultaneously focus on the relationship between plasma concentrations and adverse reactions (e.g., delayed neutropenia, hypogammaglobulinemia, progressive multifocal leukoencephalopathy (Yuan et al., 2020)). The ultimate goal is to explore individualized dosing strategies based on plasma concentration monitoring to optimize treatment response and avoid both ineffective and excessive exposure.

## Discussion

RTX efficacy in glomerular diseases is governed by a complex exposure-response relationship, not merely dosage. Its unique pharmacokinetic properties, particularly target-mediated drug disposition and accelerated clearance due to early B-cell load, directly impact effective drug exposure (Hartinger et al., 2022). This clearance mechanism is further modulated by disease immune status, renal damage, and intrinsic patient characteristics (Golay et al., 2014). RTX’s core pharmacodynamic effect involves inducing deep and sustained B-cell depletion. However, individual variations in B-cell kinetics and the absence of disease-specific markers (e.g., PLA2R in membranous nephropathy versus minimal change disease or lupus nephritis) complicate its exposure-response relationship. A deeper understanding of these dynamic processes and their interactions is thus crucial for precise, individualized treatment of glomerular diseases.

RTX exhibits complex pharmacokinetic properties. As a large monoclonal antibody, its clearance is notably time-dependent, primarily driven by target-mediated drug disposition (Cartron et al., 2007). During initial therapy, a high B-cell load provides abundant drug targets. RTX rapidly binds to these B cells, leading to accelerated early drug elimination and a sharp decline in plasma concentration (Golay et al., 2014). This significantly impacts initial RTX exposure, thereby hindering the effective establishment of its exposure-response relationship.

As B cells become effectively depleted, the number of circulating targets sharply decreases, significantly attenuating the target-mediated drug disposition effect. Consequently, RTX clearance slows, its half-life extends, and it enters a slower elimination phase (Golay et al., 2014). This dynamic clearance pattern highlights the limitations of fixed-dose regimens. Post-B-cell depletion, drug exposure may become excessive, increasing the risk of adverse events. Conversely, if B-cell clearance is incomplete, drug exposure might be insufficient, potentially leading to treatment failure or drug resistance. Therefore, real-time pharmacokinetic monitoring and dose adjustments based on B-cell kinetics are essential to optimize the exposure-response relationship and achieve personalized medicine.

The core pharmacodynamic effect of RTX lies in inducing profound and durable depletion of peripheral CD20^+^ B lymphocytes (Beum et al., 2011). Adequate drug exposure is essential for complete B-cell depletion and its maintenance, with depletion depth and duration directly correlating with clinical remission rates (Tedesco et al., 2022). However, the mechanisms and clinical implications of B-cell depletion differ significantly across glomerular diseases, influencing their respective exposure-response relationships.

In MCD, initial B-cell burden is relatively low (Zhong et al., 2025). Nevertheless, inter-individual pharmacokinetic variability persists. Notably, high-grade proteinuria may further accelerate the early, time-dependent clearance of RTX. This presents a challenge to achieving the adequate drug exposure required for therapeutic efficacy and contributes to greater pharmacokinetic uncertainty. Although the pathogenesis of MCD is primarily attributed to T-cell abnormalities and podocyte injury, anti-Nephrin autoantibodies produced by B-cells are a key component of its pathogenic mechanism. RTX depletes these specific B-cells, thereby reducing anti-Nephrin autoantibody levels and mitigating podocyte injury. On the other hand, RTX may indirectly modulate T-cell function (e.g., by impacting the role of B-cells as antigen-presenting cells), thus improving podocyte function and reducing proteinuria (Zhong et al., 2025). This indirect mechanism of action implies that the exposure-response relationship in MCD may be more complex and exhibit a time lag, necessitating prolonged drug exposure for pharmacodynamic effects to manifest. Consequently, the monitoring of B-cell kinetics requires enhanced sensitivity and continuity to capture its subtle changes.

Conversely, in LN, B-cell depletion plays a more direct and pivotal role. B cells not only produce autoantibodies but also serve as potent antigen-presenting cells and cytokine producers. Thus, effective B-cell depletion directly suppresses autoimmune responses, reducing inflammation and tissue damage (Zisa et al., 2024; Houssiau, 2014). This pleiotropic action makes the exposure-response in LN a complex, multidimensional dynamic process, requiring comprehensive assessment of B-cell depletion’s impact on the entire immune network.

Furthermore, B-cell depletion is not the sole therapeutic endpoint; post-depletion B-cell kinetics, particularly repopulation rate and phenotype, are crucial. Rapid B-cell repopulation, especially of memory B cells, often predicts disease relapse (Lan et al., 2024; Teisseyre et al., 2021a), thereby undermining established exposure-response relationships. Individual B-cell kinetic variations, influenced by host genetics, disease activity, and potential B-cell sanctuaries, introduce nonlinearity and uncertainty into the exposure-response. Therefore, optimal strategies should prioritize continuous monitoring of B-cell repopulation dynamics and timely re-treatment to maintain effective exposure and response.

In MN, PLA2R dynamics offer a precise insight into the RTX exposure-response relationship (Seitz-Polski et al., 2018). Post-RTX, serum anti-PLA2R antibody levels decline, often lagging B-cell depletion but correlating strongly with proteinuria remission and long-term prognosis (Seitz-Polski et al., 2018). This PLA2R reduction directly reflects effective B-cell control and suppressed autoantibody production. Thus, sustained anti-PLA2R antibody decline serves as a dynamic marker linking effective RTX exposure to B-cell control and subsequent immunological and clinical responses (Boyer-Suavet et al., 2020). This understanding positions PLA2R kinetics as crucial for therapeutic drug monitoring in MN, enabling individualized treatment strategies and optimized exposure-response.

However, for MCD and LN, such highly specific and quantifiable biomarkers like PLA2R are absent due to their distinct pathogenesis and broader immune dysregulation. This lack of a key pharmacodynamic marker complicates understanding and predicting the RTX exposure-response in these conditions. Clinicians must rely on a range of non-specific indicators (e.g., proteinuria, renal function, C3/C4, anti-dsDNA antibody titers) (Hartinger et al., 2022) to indirectly assess PD effects and disease progression. This increases the complexity of evaluating treatment adequacy and optimizing dosing, underscoring the urgent need for novel disease-specific PD markers to develop precise exposure-response models.

All in all, RTX efficacy in glomerular diseases relies on a complex exposure-response relationship, intricately modulated by B-cell kinetics, PLA2R dynamics (critical in MN), and time-dependent clearance. Early accelerated clearance significantly limits initial and sustained drug exposure, forming a bottleneck for effective B-cell depletion. Moreover, individual variations in B-cell depletion depth, duration, and repopulation rates directly determine treatment durability and clinical outcome stability.

## Future research directions and clinical translation

Although research on RTX in glomerular diseases has made significant progress, clearly demonstrating a close relationship between its pharmacokinetic characteristics and clinical efficacy, its clinical application still faces challenges in precision medicine due to pharmacokinetic heterogeneity. The majority of current evidence derives from small-sample retrospective studies or population-average analyses, which fail to fully elucidate the association between individual exposure variability and treatment outcomes. Of particular concern is that the clinically referenced effective concentration threshold (e.g., 10 μg/mL) is primarily derived from *in vitro* studies or models of other autoimmune diseases. The direct extrapolation of this threshold to the specific pathophysiological context of glomerular diseases lacks direct supporting evidence and should be regarded more as a reference starting point for pharmacological investigation.

Key future research directions include: (1) conducting prospective, disease-specific concentration–response studies to establish evidence-based target exposure windows; and (2) developing pharmacokinetic prediction models that integrate multidimensional clinical features.

## Conclusion

The pharmacokinetics of RTX in glomerular diseases exhibit significant heterogeneity, modulated by multiple factors including proteinuria-mediated renal loss, ADA formation, FcRn receptor competition, and disease-specific B-cell burden. This results in characteristic exposure patterns across different disease types: MN patients have an approximate half-life of 11.5 days; MCD patients show a slightly longer half-life (around 14.6 days); whereas LN patients experience the most rapid clearance due to high B-cell burden and an inflammatory state, leading to a significant decline in plasma concentrations by the second month. Adequate and sustained drug exposure correlates positively with the depth of B-cell depletion and clinical remission. However, excessive exposure might diminish efficacy through trogocytosis-mediated CD20 antigen stripping and can induce severe adverse reactions, including infections.

Clinical practice necessitates a shift from “fixed-dose” to “'exposure-based individualized dosing”. Based on the aforementioned studies, we recommend monitoring RTX plasma concentrations at 2–3 months post-administration for MN; at 2–3 months for single-dose MCD regimens, and 4–6 months for multi-dose MCD regimens; and prior to 2 months for LN. This allows for timely identification of underexposure (<10 μg/mL) and subsequent administration of rescue doses. For refractory or ADA-positive patients, consider switching to obinutuzumab or combining with calcineurin inhibitors to reduce proteinuria and optimize drug exposure. By implementing disease-specific monitoring strategies and individualized interventions, we can maximize clinical benefits while mitigating the risks of ineffective or excessive exposure, thereby achieving true precision immunotherapy.
